# Low-field 0.55 T MRI evaluation of the fetus

**DOI:** 10.1007/s00247-023-05604-x

**Published:** 2023-03-08

**Authors:** Skorn Ponrartana, HaiThuy N. Nguyen, Sophia X. Cui, Ye Tian, Prakash Kumar, John C. Wood, Krishna S. Nayak

**Affiliations:** 1 Department of Radiology, Children’s Hospital Los Angeles, Los Angeles, CA USA; 2 Siemens Medical Solutions, USA, Inc, Los Angeles, CA USA; 3 Department of Electrical and Computer Engineering, University of Southern California, Los Angeles, CA USA; 4 Division of Pediatric Cardiology, Children’s Hospital Los Angeles, Los Angeles, CA USA

**Keywords:** Fetal, Low-field, Magnetic resonance imaging, Real-time imaging, Safety

## Abstract

Fetal magnetic resonance imaging (MRI) is an important adjunct modality for the evaluation of fetal abnormalities. Recently, low-field MRI systems at 0.55 Tesla have become available which can produce images on par with 1.5 Tesla systems but with lower power deposition, acoustic noise, and artifact. In this article, we describe a technical innovation using low-field MRI to perform diagnostic quality fetal MRI.

**Supplementary Information:**

The online version contains supplementary material available at 10.1007/s00247-023-05604-x.

## Introduction

Magnetic resonance imaging (MRI) has evolved into an important tool in the diagnosis of fetal abnormalities [1]. As an adjunct to prenatal ultrasound, MRI improves accuracy in diagnosis through the added benefit of superior soft tissue contrast, larger field-of-view, and improved visualization of the fetus in the setting of oligohydramnios, maternal obesity, or difficult fetal position [2]. Fetal MRI has generally been performed clinically on 1.5 T systems but in the past decade, there has been a shift to 3 T systems, which offer improved signal-to-noise that can be leveraged to improve spatial and/or temporal resolution, albeit with the trade-off of increased artifacts, higher radiofrequency specific absorption rate (SAR), and higher acoustic noise [3].

Recently, the development of low-field-strength 0.55 T MRI systems represent a major break from this trend. Compared to conventional 1.5 T and 3 T systems, low-field MRI offers superior magnetic field (B0) and radiofrequency transmit field (B1 +) homogeneity, shorter T_1_ and longer T_2_^*^, more flexible pulse sequence design due to decreased susceptibility artifacts and markedly lower SAR, and lower acoustic noise improving patient comfort [4]. In this article, we demonstrate a technical innovation of using low-field MRI to perform a diagnostic-quality fetal MRI.

## Description

### Experimental methods

All study images were collected on a whole-body 0.55 T system (prototype MAGNETOM Aera XQ, Siemens Healthineers, Erlangen, Germany) equipped with high-performance shielded gradients (45 mT/m amplitude, 200 T/m/s slew rate). All participants were imaged in supine position. Data were collected using a 6-channel body array positioned over the lower pelvic area (anterior) and 6 to 9 elements from a table-integrated 18-channel spine array (posterior). Eight healthy gravid volunteers with normal pregnancies (age 31–42 years, gestational age 24–34 weeks) were scanned under a protocol approved by our Institutional Review Board, after providing written informed consent.

### Fetal MRI protocol

The standard fetal MRI protocol utilizes rapid acquisition sequences to limit the effects of fetal motion. Generally, these include T2-weighted half-Fourier single-shot turbo spin echo (HASTE) and balanced steady-state free precession (bSSFP) images in multiple planes to depict anatomy as well as T1-weighted gradient-echo (GRE) sequences to identify specific tissue/fluid characteristics such as fat, hemorrhage, or meconium [5, 6]. Diffusion-weighted imaging (DWI), hydrography, and cine imaging can also provide added value.

We hypothesized that the advantages of low-field MRI could be used to improve the HASTE and bSSFP sequences. At conventional field strengths (≥ 1.5 T), both are limited by SAR. HASTE is performed with suboptimal refocusing flip angles, and bSSFP is performed with suboptimal imaging flip angles. SAR constraints are virtually eliminated at 0.55 T, making it possible to use 180° refocusing pulses for HASTE, and contrast-optimal flip angles for bSSFP. The relaxed off-resonance constraints of this system also enable lengthening of readout and substantially improved sampling efficiency (roughly threefold, compared to 1.5 T).

**Fig. 1:**
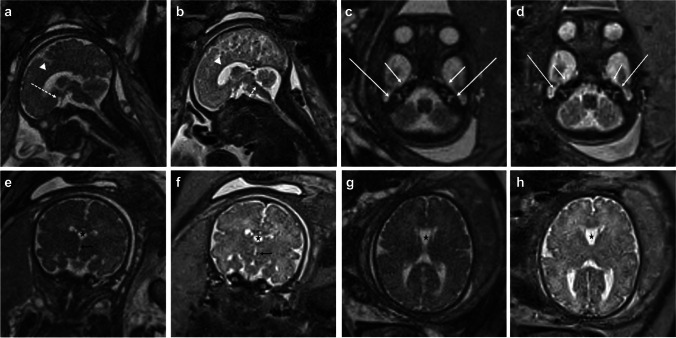
34-week gestational age fetus. Sagittal, axial, and coronal representative images of the fetal brain with half-Fourier single-shot turbo spin echo (HASTE) and balanced steady-state free precession (bSSFP). Sagittal bSSFP (**a**) and HASTE (**b**) images show normal midline structures including the corpus callosum (arrowhead), optic chiasm/nerve (long dashed arrow), and 4th ventricle (short dashed arrow). Axial bSSFP (**c**) and HASTE (**d**) images at the level of the inner ears partially resolves the cochlea (short solid arrow) and semi-circular canals (long solid arrow). Coronal bSSFP (**e**) and HASTE (**f**) images through the frontal horns demonstrate a normal cavum septi pellucidi (star), third ventricle (black arrow), and normal sulcal/gyral pattern. Axial bSSFP (**g**) and HASTE (**h**) images through the lateral ventricles show a normal cavum septi pellucidi (star), lateral ventricles, and normal sulcal/gyral pattern

**Fig. 2:**
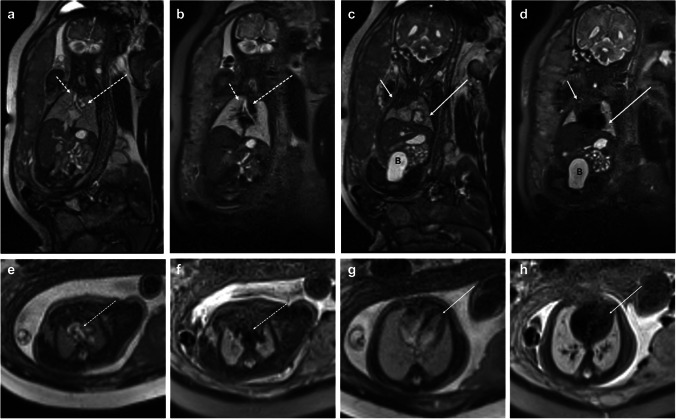
32-week gestational age fetus. Coronal balanced steady-state free precession (bSSFP) (**a**) and half-Fourier single-shot turbo spin echo (HASTE) (**b**) images show a normal fluid-filled tracheobronchial tree (short dashed arrow), mediastinal vascular structures (long dashed arrow), liver, stomach, and bowel. Coronal bSSFP (**c**) and HASTE (**d**) images more anterior demonstrate a normal thymus (short solid arrow) and left ventricle (long solid arrow), and bladder (B) as well. Axial bSSFP (**e**) and HASTE (**f**) images at the level of the great vessels and axial bSSFP (**g**) and HASTE (**h**) show bright and black blood images of flowing blood into the heart (long solid arrow) and great vessels (long dashed arrow) and contrast with the fluid-filled lungs

A list of typical scan parameters is summarized in Table 1. Representative images of the fetal brain and body using low-field MRI are shown in (Figs. 1 and 2).

**Table 1:** Key imaging parameters for the 0.55 T fetal MRI protocol as implemented on a 0.55 T scanner

Sequence		TR (ms)	TE (ms)	FA (deg)	BW (Hz/Px)	Slice thickness (mm)	Slice gap (mm)	Avg	Number of slices	FOV (mm^2^)	Acquisition matrix	Voxel size (mm^3^)	Partial Fourier	Acquisition time	
														Per slice (s)	Total
HASTE	Body	2000	98	180	251	3	0	1	30	350 × 350	224 × 224	1.6 × 1.6 × 3.0	4/8	2.0	1:00
	Brain	2000	98	180	251	3	0	1	30	300 × 300	224 × 224	1.3 × 1.3 × 3.0	4/8	2.0	1:00
bSSFP	Body	6.02	3.01	120	250	3	0	1	30	350 × 350	192 × 192	1.8 × 1.8 × 3.0	None	1.5	0:45
	Brain	6.02	3.01	120	250	3	0	1	30	300 × 300	192 × 192	1.6 × 1.6 × 3.0	None	1.5	0:45
Real-time cine		570	1.82	70	558	10	-	1	1	350 × 350	128 × 128	2.8 × 2.8 × 10	None	0.6	-
T1w GRE		113	2.43	60	250	4	0	1	20	295 × 350	138 × 192	1.8 × 1.8 × 4.0	None	-	0:16
DWI (*b* = 0.700)		2500	74	-	1510	4	0	1	20	294 × 330	82 × 92	3.6 × 3.6 × 4.0	6/8	-	0:30

**Fig. 3:**
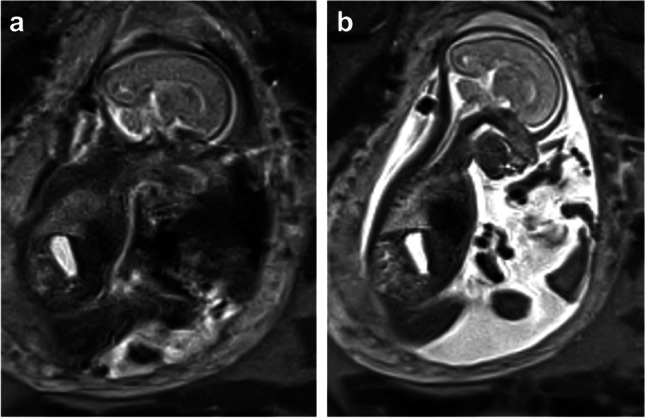
24-week gestational age fetus. Sagittal whole body half-Fourier single-shot turbo spin echo images were performed with a TR of 1400 ms (**a**) and TR of 2000 ms (**b**). At TR of 1400 ms, amniotic fluid was markedly saturated, limiting evaluation of the umbilical vessels and extremities. The TR was subsequently increased until reaching an optimal value of 2000 ms

**Fig. 4:**
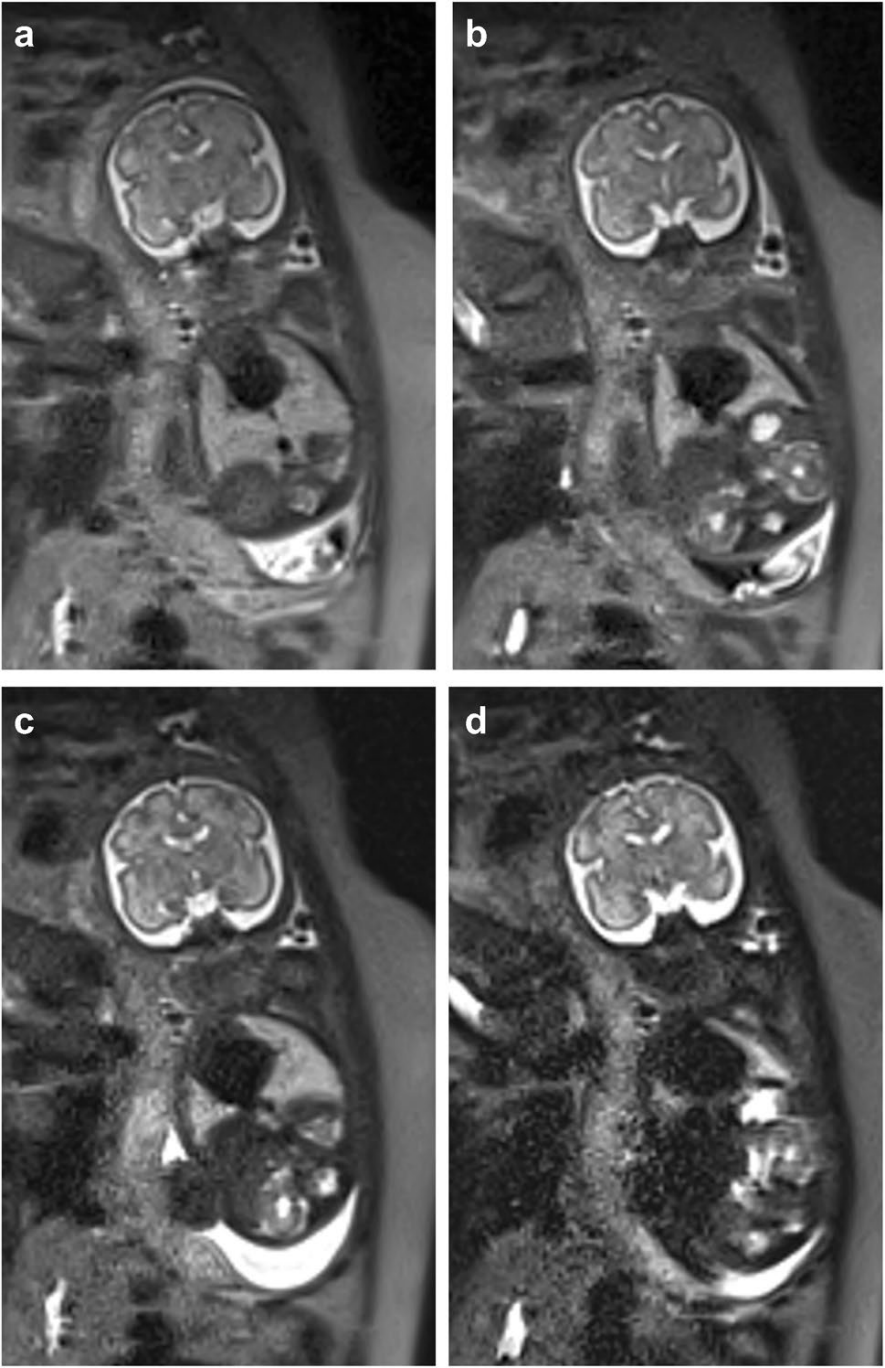
28-week gestational age fetus. Coronal whole body T2 half-Fourier single-shot turbo spin echo images were performed with a TE of 77 ms (**a**) 98 ms (**b**) 181 ms (**c**) and 272 ms (**d**). Signal intensity decreased at higher TE, as delineated by the higher noise in the fetal body on (**d**). However, contrast resolution slightly increased at higher TE, best seen at the gray-white matter junction of the brain

*HASTE* was used to produce the single-shot T2-weighted contrast. The half-Fourier acquisition effectively reduces echo train length and therefore limit the intra-scan motion. Imaging parameters were slice thickness of 3 mm, FOV 350 × 350 mm^2^, and matrix resolution of 224 × 224 resulting in a voxel size of 1.6 × 1.6 × 3.0 mm^3^, comparable to that of typical 1.5 T fetal SST2W protocols which have 1 × 1 to 2 × 2 mm^2^ in-plane spatial resolution with slice thickness between 2 and 4 mm [6]. A lower bandwidth of 250 Hz/Px compared to that of a typical 1.5 T protocol (300 to 700 Hz/Px) was used to increase SNR. HASTE at 1.5 T typically uses suboptimal refocusing flip angles of 90°-120° to limit SAR deposition. We increased this value to 180° to improve SNR, given that SAR is 7.5-fold lower at this field strength. To determine the optimal TR and TE at 0.55 T, multiple different TR and TE values were compared and ultimately a TR of 2000 ms and TE of 98 ms were selected (Figs. 3 and 4).

**Fig. 5:**
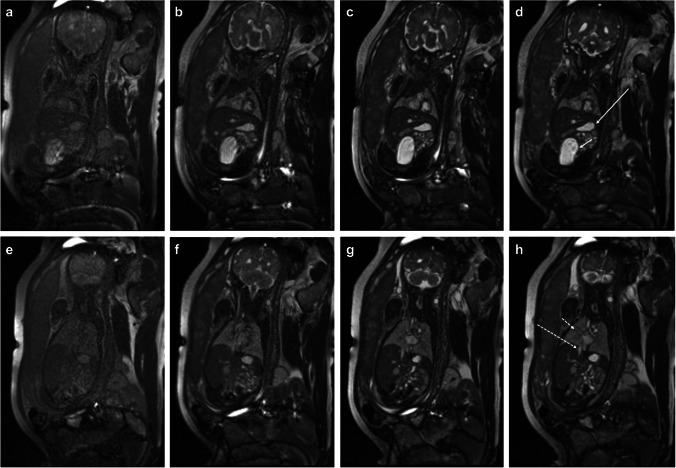
34-week gestational age fetus. Coronal whole body balanced steady-state free precession images at increasing flip angles, 30° (**a**, **e**), 60° (**b**, **f**), 90° (**c**, **g**), and 120° (**d**, **h**). As flip angles increased, there was improved signal to noise and contrast resolution, in particular flowing blood in the inferior vena cava (long dashed arrow) versus fluid filled lungs (short dashed arrow) versus static fluid in the stomach (long solid arrow) and bladder (short solid arrow)

*Single-shot bSSFP* employs a flip angle of 60° at 1.5 T due to SAR limits. We selected a flip angle of 120° because it provided optimal image contrast and SNR efficiency (Fig. 5). The minimum TR/TE given the other imaging parameter was used, namely 6.0/3.0 ms. We imaged with a voxel volume equivalent to our clinical scans at 1.5 T (9–10 mm^3^) and adjusted acquisition parameters to balance SNR and resolution. We settled on voxel size of 1.8 × 1.8 × 3.0 mm^3^ or volume of 9.7 mm^3^. Single-shot bSSFP was also used for real-time localization and dynamic imaging at a frame rate of 0.6 s per image.

*Diffusion weighted imaging (DWI)* was acquired with single-shot EPI readout with *b*-value = 0 and 700.

**Fig. 6:**
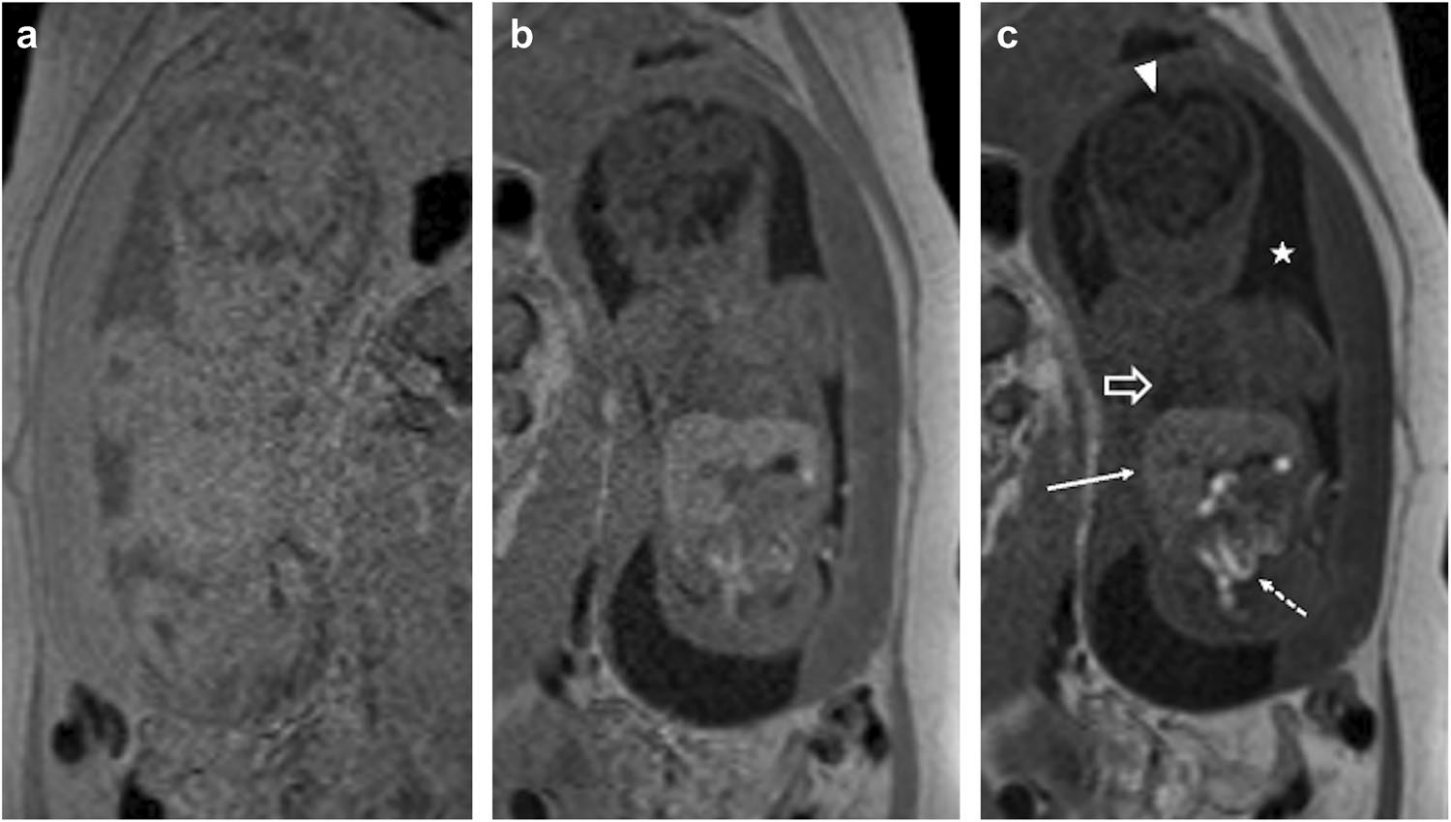
25-week gestational age fetus. Coronal spoiled gradient-recalled echo images at increasing flip angles, 15° (**a**), 30° (**b**), and 60° (**c**), show improved contrast of short T1 structures such as liver (long solid arrow) and meconium filled bowel (short dashed arrow), as well as improved but subtle gray-white matter differentiation. Additionally, there is increased distinction of tissues with longer T1, such as amniotic fluid (star), fluid-filled lungs (open arrow), and cerebral spinal fluid (arrowhead)

*Spoiled gradient echo (GRE)* was used to produce the T1-weighted contrast to identify features such as subacute bleeding, calcification, lipoma, meconium, and hemorrhage. Single echo spoiled gradient echo imaging using a flip angle of 60° provided the best image contrast (Fig. 6).

#### Acoustic noise measurements

In one subject, sound pressure level was measured using a sound level meter (B&K Sound Level Meter/Analyzer Type 2250, Brüel & Kjær, Nærum, Denmark) and an MRI-compatible microphone (B&K Prepolarized Free-field Microphone Type 4189, Brüel & Kjær, Nærum, Denmark) that was placed at the MRI bore opening. The absolute sound level was 86.2–86.4 dB during localizer sequences, 79.2–80.3 dB during HASTE, and 86.2–87.5 dB during bSSFP. Variations within each range can be attributed to changes in slice/slab orientation. The absolute sound levels are comparable with a study by Rusche et al. that also evaluated acoustic noise at 0.55 T, with the levels ranging from 83.7 to 86.3 dB [7]. However, these levels are lower than reported measures in both 1.5 T and 3.0 T, which ranged from 101.8 to 111.7 dB and 125.7 to 130.7, respectively [8].

#### SAR measurements

At 0.55 T, the average SAR for HASTE acquisitions is 0.31 W/kg and 0.51 W/kg for bSSFP acquisition, compared with 1.8 W/kg and 1.4 W/kg at 1.5 T, respectively.

## Discussion

Our work shows that low-field MRI is a viable alternative for current clinical fetal MRI. To our knowledge, low-field MRI systems have never been applied to fetal imaging. By optimizing product HASTE, bSSFP, GRE, and DWI sequences to low-field MRI, we were able to generate diagnostic quality fetal MRI protocol.

While the SNR is expected to be lower at 0.55 T compared with 1.5 T and 3 T, we were able to recoup those differences by optimizing the intrinsic advantages of low-field MRI. For example, because of the lower SAR at 0.55 T, we were able to go beyond the normal flip angle, TR, and slice number constraints at higher field strengths to improve image quality. Additionally, at lower magnetic field strengths, there is a shorter tissue T1, longer T2, and longer T2, which decreases the scan time. In addition to helping with motion artifact, we were able to adjust other parameters that lead to increased scan time, such as flip angle, echo train length, and signal averaging. Finally, specifically for bSSFP at 0.55 T, we were able to obtain improved image contrast because T2/T1 is much higher at 0.55 T than at 1.5 T (almost twofold).

Low-field MRI has distinct advantages compared to clinical 1.5 T and 3 T scanners. Real-time 3D imaging is feasible, allowing the MRI operator to track the fetus as it moves and capture diagnostic quality images during quiescence, which will dramatically shorten exam time, increase throughput, and improve MRI diagnosis of fetal limb and cardiac abnormalities. SAR and acoustic noise are reduced, making the exam safer for both mother and fetus. There is a lower cost and more modest siting requirements for low-field MRI, which needs less helium, space, and flooring requirements, thereby improving accessibility for smaller hospitals and developing countries. Finally, because of decreased superconducting wire requirements, the bore is wider for low-field MRI scanners (70 cm for the 0.55 T prototype Aera (modified MAGNETOM Aera Siemens Healthineers, Erlangen, Germany) and 80 cm for the Free.Max (0.55 T MAGNETOM Free.Max, Siemens Healthineers, Erlangen, Germany)) compared with 60 cm in most traditional MRI scanners, which will improve patient comfort.

### Current challenges

An initial challenge for low-field MRI will be its availability. Low-field MRI is a new technology and therefore will take time before there is widespread access beyond academic centers. However, the first FDA-approved low-field systems (0.55 T MAGNETOM Free.Max, Siemens Healthineers, Erlangen, Germany) have been deployed since 2021, meaning many more sites are beginning to acquire low-field MRI capabilities.

A more significant challenge of low-field MRI is its inherently lower SNR due to reduced polarization (compared to 1.5 T and 3 T). One would think this would lead to difficulty achieving diagnostic quality. However, as mentioned previously, this effect was largely compensated by optimizing MRI imaging parameters. Additional future techniques that can be used at this field strength to improve SNR include novel data sampling, novel coil arrays, novel reconstruction and processing (including artificial intelligence), and slice-to-volume registration tools.

Another challenge is concomitant field effects, which cause artifacts at the periphery of very large fields-of-view, and when imaging far from isocenter. HASTE and bSSFP are particularly sensitive to these effects as they are sensitive to non-linear spatially varying phase across the subject. In many cases, these effects can be predicted with high accuracy and compensated during reconstruction, with added computation complexity [9]. In this work, we did not observe any significant ill effects from concomitant fields; however, they will be an important consideration when developing more advanced data sampling approaches or pulse sequences.

### Future directions

Before full acceptance of the innovation can occur, further research should be performed to validate the diagnostic quality of low-field fetal MRI with current standard of care 1.5 T and 3 T using larger sample sizes. Another line of research could focus on optimizing fetal cardiac MRI, since bSSFP images are useful for cardiac images and can be better optimized at low field. Finally, the full benefits of real-time fetal MRI should be explored. With real-time MRI, multiplanar acquisitions could be obtained over a time period with retrospective selection of 2D slices to evaluate fetal motion, swallowing, and cardiac activity, similar to ultrasound (Online Supplementary Material 1–3). This approach may benefit from adopting slice-to-volume registration tools that have been developed by the research community.

## Conclusion

In conclusion, low-field MRI is a promising innovative technique for fetal imaging that may provide similar diagnostic quality images as current clinically standard 1.5-T fetal MRI. However, compared to currently established fetal MRI techniques, low-field MRI has additional distinct advantages including low SAR, low acoustic noise, and real-time imaging capabilities. Future research is needed to compare head-to-head the diagnostic performance of low field with both 1.5 T and 3 T fetal MRI and explore new applications for fetal cardiac MRI and real-time fetal MRI imaging.

## Supplementary Information

Below is the link to the electronic supplementary material.

Supplementary file1 (AVI 121861 KB)

Supplementary file2 (AVI 25603 KB)

Supplementary file3 (AVI 66564 KB)
